# Effect of Media Opacity on Retinal Nerve Fiber Layer Thickness Measurements by Optical Coherence Tomography

**Published:** 2010-07

**Authors:** Dae Woong Lee, Joon Mo Kim, Ki Ho Park, Chul Young Choi, Jung Gon Cho

**Affiliations:** 1 Department of Ophthalmology, Sungkyunkwan University School of Medicine, Kangbuk Samsung Hospital, Seoul, Korea; 2 Department of Ophthalmology, Seoul National University College of Medicine, Seoul, Korea; 3 Yebon Eye Clinic, Seoul, Korea

**Keywords:** Ocular Media Opacity, Optical Coherence Tomography, Signal Strength

## Abstract

**Purpose:**

To assess the effect of ocular media opacity on retinal nerve fiber layer (RNFL) thickness measurements by optical coherence tomography (OCT).

**Methods:**

In this prospective, non-randomized clinical study, ocular examinations and OCT measurements were performed on 77 cataract patients, 80 laser refractive surgery patients and 90 patients whose signal strength on OCT was different on two consecutive measurements. None of the eyes had preexisting retinal or optic nerve pathology, including glaucoma. Cataracts were classified according to the Lens Opacity Classification System III (LOCS III). All eyes were scanned with the Stratus OCT using the Fast RNFL program before and three months after surgery. Internal fixation was used during scanning and all eyes underwent circular scans around the optic disc with a diameter of 3.4 mm.

**Results:**

Average RNFL thickness, quadrant thickness and signal strength significantly increased after cataract surgery (P<0.05). Cortical and posterior subcapsular cataracts, but not nuclear cataracts, had a significant influence on RNFL thickness measurements (P<0.05). There was no significant difference between OCT parameters before and after laser refractive surgery. In eyes for which different signal strengths were observed, significantly larger RNFL thickness values were obtained on scans with higher signal strengths.

**Conclusion:**

OCT parameters are affected by ocular media opacity because of changes in signal strength; cortical cataracts have the most significant effect followed by posterior subcapsular opacities. Laser refractive procedures do not seem to affect OCT parameters significantly.

## INTRODUCTION

The diagnosis of glaucoma and determination of its progression are made through a combination of clinical examination, static perimetry, stereoscopic disc photography, and red free photography.^1^ Stereo disc photography and red free photography are sensitive tools for identifying early progression, but the subjectivity involved in interpretation of these examination techniques and the poor image quality seen in patients with ocular media opacity makes them less than ideal.^2^,^3^ There is a steep rise in the prevalence of ocular media opacities, including cataracts, and glaucoma with increased age.

Optical coherence tomography (OCT) is a high-resolution, cross-sectional imaging technique that allows in vivo measurement of the retinal nerve fiber layer (RNFL).^4^ OCT images are representations of tissue structure based on intensity of light backscattering, which is highly dependent on the optical properties of the structure. Altered ocular media transparency reduces the signals reflected from the retina and may therefore affect image quality. According to previous studies, RNFL thickness measurements by Stratus OCT are affected by a variety of factors, such as cataracts, vitreous opacity, or alterations in corneal refractive index.^4^–^8^ OCT is widely used to diagnose both retinal and optic nerve disease, and the influence of media opacities including cataracts and vitreous opacities (asteroid hyalosis, silicone oil), on OCT image quality has been previously studied.^9^–^16^

In this study, we investigated the effect of ocular media opacity on RNFL measurements determined by OCT in patients undergoing cataract or excimer laser refractive surgery, and in cases with different signal strengths on consecutive OCT images.

## METHODS

### Subject Recruitment

We recruited and prospectively evaluated a total of 89 out of 218 patients scheduled for cataract surgery, and 80 excimer laser refractive surgery candidates from September 2005 to April 2006. In addition, 90 eyes of 668 subjects who were scanned twice consecutively on the same day were selected, for which the difference in signal strength (SS) was one unit. Cataract patients were eligible if lens opacities were the only media opacities present in the eye. Lenticular opacities were classified as nuclear, cortical, or posterior subcapsular based on the highest score using the LOCS III system.^17^ If the score was greater than NO3-NC3 but less than C3 and P3, the cataract would be categorized as nuclear. If the score was greater than C3, the opacity was classified as cortical and if it was greater than P3, it was considered to be posterior subcapsular. Laser refractive surgery patients were myopic but were free of any ocular or systemic disease. Every candidate who was referred for cataract or refractive surgery was considered for enrollment into the study except for those who presented with the exclusion criteria.

Exclusion criteria were corneal and vitreous opacities, pre-existing retinal or optic nerve pathology, history of glaucoma and previous ocular surgery. Informed consent was obtained from each participant and the study was approved by the local institutional review board and adhered to the tenets of the declaration of Helsinki.

### Methods

Subjects underwent the following examinations before and three months after cataract or laser refractive surgery: a review of medical and ophthalmic history and a precise ophthalmic examination including manifest refraction, best corrected visual acuity (BCVA), intraocular pressure (IOP), slit lamp biomicroscopy and fundus examination.

RNFL thickness measurement was performed using the Stratus OCT (software version 4.0.1, Carl Zeiss Meditec, Dublin, USA) by a single experienced technician. All pupils were dilated with one drop of 2.5% phenylephrine hydrochloride and one drop of 0.5% tropicamide. The operator applied artificial tears (Hyalein, Santen Pharmaceuticals, Osaka, Japan) for tear film stability before the scan. Twenty minutes after instillation of mydriatics and adequate pupil dilation, a peripapillary fast RNFL thickness scan was performed on each subject with a preset scan diameter of 3.4 mm centered on the optic disc. Centration of scans was verified by direct observation of the disc on the screen. An internal fixation target was provided during image acquisition. The fast RNFL thickness scanning protocol consists of 3 scans. Each scan comprises of 256 measurement points along a circular path with a radius of 1.73 mm. The software presents average values of these 3 scans. Only complete scans of adequate quality which were free of artifacts and had signal strength (SS) of 5 or more (maximum, 10), were used for data analysis. OCT scans were performed before surgery and repeated three months after surgery.

All cataract surgeries were sutureless phacoemulsificaton procedures performed by the same surgeon (JMK) through a 2.8 mm temporal clear corneal incision together with implantation of a single-piece acrylic posterior chamber intraocular lens (Acrysof SA60AT, Alcon Labratories, Fort Worth, USA).

All laser refractive procedures were laser-assisted subepithelial keratectomy (LASEK) performed by a single surgeon (JGC) utilizing the Mel 80TM excimer laser system (Aesculap-Meditec, Jena, Germany).

### Data Analysis

To evaluate postoperative changes in image quality, pre- and postoperative signal strengths (SS) were compared. Differences in pre- and postoperative RNFL thickness measurements were assessed using mean and quadrant RNFL thicknesses. To study the effect of SS on RNFL thickness measurements, we analyzed OCT scans of 90 subjects with different signal strengths on two different image acquisitions. We used the paired t-test implemented in PASW 17.0 (SPSS Inc., Chicago, USA) to compare RNFL thickness and SS before and after surgery in both cataract and refractive surgery candidates. To compare RNFL thickness and SS before and after surgery among different cataract subtypes, we used linear mixed model analysis. In addition, SS and RNFL thickness before and after surgery were analyzed with a linear mixed model to investigate changes in RNFL thickness per one unit of change in SS. P-values less than 0.05 were considered as statistically significant.

## RESULTS

### Cataract Patients

Initially, 89 eyes of 89 patients were enrolled for the study, however 12 eyes were excluded due to poor preoperative image quality (SS less than 5). Eventually, 77 eyes of 77 subjects including 20 male and 57 female participants with mean age of 67.8±9.3 years were analyzed. Cataract subtypes were designated according to the predominant type of opacity and included 25 cortical (C), 30 nuclear (N) and 22 posterior subcapsular (P) opacities. There was no statistically significant difference in BCVA among the cataract subgroups (P > 0.05).

RNFL thickness and SS prior to and after cataract surgery are detailed in Table 1. Preoperatively, average RNFL thickness was 93.1±16.6 μm which increased to 97.9±17.9 μm postoperatively (P< 0.001). RNFL thickness increased significantly in all four quadrants after cataract surgery (all P values < 0.05). Mean preoperative SS was 6.0±1.3 which improved to 6.8±1.5 postoperatively (P<0.001).

RNFL analyses according to cataract subtypes are shown in Table 2. The observed increase in mean SS and average RNFL thickness after cataract surgery was significant in eyes with cortical and posterior subcapsular cataracts (P<0.05). However, there was no significant change in SS or RNFL thickness following surgery in eyes with nuclear cataracts.

**Figure 1:**
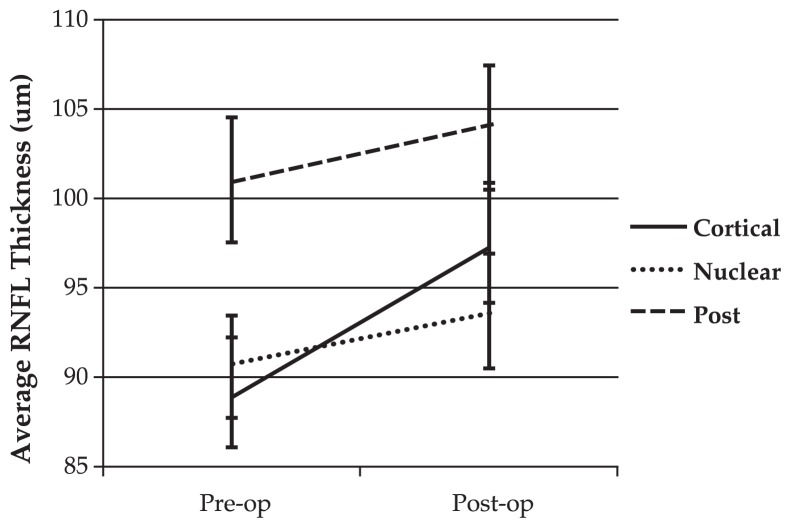
Average retinal nerve fiber layer (RNFL) thickness before and after phacoemulsification. Cataract surgery significantly increased average RNFL thickness in different categories by different amounts (P=0.001, linear mixed model).

**Figure 2:**
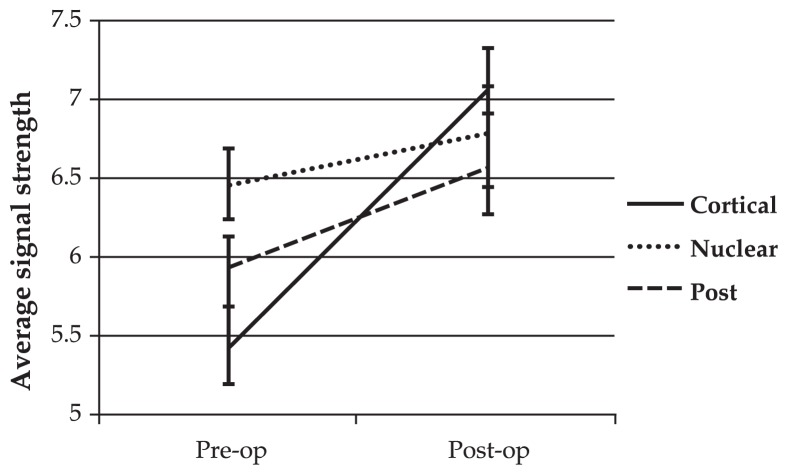
Mean signal strength before and after phacoemulsification. Cataract surgery significantly increased signal strength in different categories by different amounts (P=0.020, linear mixed model).

Linear mixed model analysis revealed that the rate of change in RNFL thickness and SS differed significantly (P<0.05) among the three groups before and after surgery (Figures 1 and 2). This analysis also revealed an association between changes in SS and measured RNFL thickness after cataract surgery. The estimated RNFL thickness change per unit of change in SS after cataract surgery was 4.6±0.9 (P<0.001).

### Laser Refractive Surgery Patients

Eighty eyes of 80 patients who underwent excimer laser refractive surgery were evaluated. No significant change was observed in any parameter (average RNFL thickness, inferior average, superior average, temporal average, nasal average and SS) before and after laser surgery (all P values > 0.05, Table 3).

### Eyes with Variable OCT Signal Strength

Eyes with disparate SS (with a difference of one unit) on two consecutive OCT scans performed on the same day had significantly thicker average and quadrant RNFL thickness values on images with higher SS. Further details regarding this subgroup of patients are presented in Table 4.

## DISCUSSION

The current study demonstrated lower RNFL values measured by Stratus OCT in patients with cataracts prior to surgery which improved significantly following phacoemulsification. This increase was marked in eyes with cortical and posterior subcapsular opacities, which may be attributed to lower signal strength figures. We anticipated the influence of cataracts on RNFL thickness measurement because the OCT signal is composed of backscattered light from tissues and is thus affected by optical properties such as refractive index, light absorption and scatter. A decrease in ocular media transparency reduces the intensity of the reflected signal from retinal layers.^7^,^8^ It is quite likely that weaker signals, occurring in the presence of cataracts, uveitis and retinal disease, affect RNFL thickness calculation by OCT software algorithms. Ray et al^18^ reported that degraded images and artifacts occur more frequently in eyes with one or more non-retinal ophthalmic disorders than those without ocular disease.

The signal to noise ratio (SNR), used in previous versions of OCT software, is determined by the ratio of signal amplitude to standard deviation of noise. The limitation of SNR is that it takes into account only the A-scan demonstrating the strongest signal and does not consider the distribution of signals throughout scan images.^19^,^20^ Signal strength is used as an image quality parameter in Stratus OCT. Signal strength is a combination of image quality (SNR) and the uniformity of signal strength among different A-scans. It has been reported that signal strength can improve the discrimination of poor images as compared to use of SNR alone.^21^ In general, greater signal strength indicates a better image and thus reflects higher confidence in the analyses. In this study, 12 cataract cases with signal strength values below 5 were discarded (according to the operation manual) to ensure accuracy of the results.

After cataract surgery, RNFL thickness measurements increased significantly. Analysis of cataract subgroups revealed that cortical and posterior subcapsular cataracts significantly affected OCT images, however, nuclear cataracts had less influence on OCT signal strength and RNFL values. Similar findings have been reported by El-Ashry et al^10^. These observations may be due to the fact that nuclear cataracts pass near-infrared light and cause minimal light scattering, while cortical and posterior cataracts scatter reflected light to a greater extent. In eyes with cortical opacities, there is a high degree of light scatter, making it difficult for the OCT beam to reach the retina and return.

The increase in quadrant RNFL thickness following cataract surgery was significant in eyes with cortical cataracts but not so with posterior subcapsular opacities. It should be noted that posterior subcapsular cataracts tend to distort OCT images more than other types of cataracts. However, eyes with a large area of posterior sub-capsular opacity were excluded from our study, because it was difficult to obtain OCT images with signal strength of more than 5.

In this study, we examined image quality by analyzing signal strength and found significantly larger RNFL values on a global scale and in all quadrants on scan images with higher SS performed in the same eye on the same day. This finding may support our observations that the increase in RNFL values following cataract surgery may be due to improved media clarity and higher SS.

According to previous studies, alterations in corneal birefringence following excimer laser refractive surgery can affect RNFL thickness measurements by scanning laser polarimetry. This has been reported to be due to alterations in corneal polarization, which is probably caused by the corneal healing process.^22^–^24^ However, the current study found no significant change in RNFL thickness or SS as measured by OCT after laser refractive surgery.

Changes in RNFL and visual field defects have been reported after LASIK.^25^,^26^ We performed laser surgery without a suction ring and observed no significant change in RNFL thickness after LASEK. These results are consistent with a previous study that also found no significant change in RNFL thickness following LASEK.^27^

In conclusion, Stratus OCT measurements of RNFL thickness are affected by ocular media opacity. We believe this effect is due to reduced SS secondary to increased light scattering by media opacities. This may result in RFNL measurements that are falsely lower in eyes with cataracts. In clinical practice, an increase in RNFL thickness can be expected after cataract surgery and we recommend obtaining new OCT images after cataract surgery to define a new baseline for patients who need glaucoma follow-up. On the other hand, corneal changes following LASEK do not seem to effect measurement of RNFL thickness by OCT, unless there is a change in corneal opacity. The Stratus OCT is still frequently used worldwide; whether or not our results are applicable to spectral domain OCT remains an open question.

## Figures and Tables

**Table 1:** Mean signal strength and RNFL thickness values before and after cataract surgery

	Preoperative	Postoperative	P value*
Signal Strength	6.0 ± 1.3	6.8 ± 1.5	< 0.001
Inferior RNFL (μm)	127.7 ± 28.2	131.2 ± 29.1	0.003
Superior RNFL (μm)	106.3 ± 24.2	112.8 ± 27.6	0.001
Nasal RNFL (μm)	66.7 ± 16.9	72.4 ± 20.0	< 0.001
Temporal RNFL (μm)	71.1 ± 17.8	74.73 ± 17.9	0.007
Average RNFL (μm)	93.1 ± 16.6	97.9 ± 17.9	< 0.001

RNFL, retinal nerve fiber layer

* Paired *t*-test

**Table 2:** Mean signal strength and RNFL thickness before and after cataract surgery in different cataract categories. Quadrant and average RNFL thickness values are expressed in μm.

	Cortical (n=25)	P value*	Nuclear (n=30)	P value*	Posterior (n=22)	P value*
Pre-SS	5.4 ± 1.2	< 0.001	6.5 ± 1.4	0.375	5.9 ± 1.1	0.034
Post-SS	7.0 ± 1.4		6.8 ± 1.7		6.6 ± 1.5	

Pre-inferior	120.0 ± 25.9	0.007	121.6 ± 24.2	0.169	144.8 ± 29.7	0.476
Post-inferior	126.6 ± 27.3		124.2 ± 27.3		146.0 ± 29.2	

Pre-superior	104.28 ± 20.0	0.003	102.1 ± 28.3	0.065	114.2 ± 21.5	0.326
Post-superior	112.6 ± 23.0		109.2 ± 26.0		117.9 ± 34.2	

Pre-nasal	60.1 ± 18.6	< 0.001	68.6 ± 17.1	0.187	70.5 ± 13.5	0.387
Post-nasal	73.6 ± 22.0		71.3 ± 19.4		72.6 ± 19.4	

Pre-temporal	72.7 ± 20.4	0.016	67.5 ± 18.6	0.182	74.4 ± 12.3	0.251
Post-temporal	77.6 ± 21.5		71.1 ± 17.4		76.4 ± 13.2	

Pre-average	89.1 ± 15.8	< 0.001	90.6 ± 15.9	0.375	101.1 ± 16.5	< 0.001
Post-average	97.5 ± 17.0		93.7 ± 18.4		104.0 ± 17.2	

RNFL, retinal nerve fiber layer; Pre, before cataract surgery; Post, after cataract surgery; SS, signal strength

* Paired *t*-test

**Table 3:** Mean signal strength and RNFL measurements before and after laser refractive surgery

	Preoperative	Postoperative	P value*
Signal strength	7.76 ± 1.54	7.90 ± 1.48	0.59
Average RNFL (μm)	102.73 ± 8.96	103.02 ± 8.84	0.627
Inferior RNFL (μm)	129.53 ± 19.60	126.66 ± 17.08	0.108
Superior RNFL (μm)	128.20 ± 16.70	131.76 ± 16.35	0.087
Nasal RNFL (μm)	66.53 ± 13.94	65.54 ± 15.55	0.549
Temporal RNFL (μm)	86.66 ± 20.28	88.05 ± 20.78	0.517

RNFL, retinal nerve fiber layer

* Paired *t*-test

**Table 4:** Difference in RNFL thickness between OCT scans of high and low signal strength

Location	Mean difference (μm)	P value*
Superior	0.749	0.026
Nasal	1.251	0.001
Inferior	0.866	0.003
Temporal	0.736	0.005
1 CH	1.274	0.009
2 CH	1.137	0.034
3 CH	1.358	0.003
4 CH	1.151	0.010
5 CH	0.719	0.103
6 CH	0.990	0.044
7 CH	1.010	0.019
8 CH	1.020	0.008
9 CH	0.823	0.004
10 CH	0.304	0.352
11 CH	0.077	0.855
12 CH	0.983	0.036
Average	0.882	0.000

RNFL, retinal nerve fiber layer; OCT, optical coherence tomography; CH, clock hour

* Paired *t*-test
